# Trials and tribulations: how we established a major incident database

**DOI:** 10.1186/s13049-017-0351-7

**Published:** 2017-01-25

**Authors:** S. E. J. Hardy, S. Fattah

**Affiliations:** 1 Emergency Medicine Department, Royal Surrey County Hospital, Guildford, Surrey England; 2 The Norwegian Air Ambulance Foundation, Drøbak, Norway; 3 Anaesthesia and Critical Care Research Group, Faculty of Health Sciences, University of Tromsø, Tromsø, Norway

**Keywords:** Major incident, Mass casualty incident, Disaster medicine, Emergency preparedness, Emergency response

## Abstract

**Objective:**

We describe the process of setting up a database of major incident reports and its potential future application.

**Method:**

A template for reporting on major incidents was developed using a consensus-based process involving a team of experts in the field. A website was set up as a platform from which to launch the template and as a database of submitted reports. This paper describes the processes involved in setting up a major incident reporting database. It describes how specific difficulties have been overcome and anticipates challenges for the future.

**Conclusions:**

We have successfully set up a major incident database, the main purpose of which is to have a repository of standardised major incident reports that can be analysed and compared in order to learn from them.

## Background

Major incidents are occurring with increasing frequency [1]. When they occur, they are chaotic and data is rarely collected during the incident. In addition, reports following a major incident are variable in quality and are non-standardised [2]. Case reports are currently the most common form of reporting on major incidents. In general, they provide little information and do not produce any evidence to foster change in our practice when it comes to the major incident response.

A pubmed search revealed 9 articles on the Paris terrorist attacks of November 2015 [3–11]. They were all effectively case reports covering different aspects of the incident from the interventions and response tactics on scene to individuals’ experiences at one particular hospital. No single article covered all aspects of the emergency response and the articles were written in such a way that data could not be collated from them. Although some of the articles identified learning points to be explored for future attacks, they did not look at the underlying reasons for failure or how to solve the problems encountered. A major incident reporting (MIR) database allows us to collect essential and standardised data on each major incident and compare them.

For the past two years, an open access MIR template has been available online at majorincidentreporting.net [12]. It is free to download the template from the webpage and free to submit and access major incident reports (Table 1).

**Table 1:** Overview of published reports on majorincidentreporting.net

Chile	
+ Prison Fire in Santiago de Chile	
Finland	
+ School shooting in Jokela	
+ Plane crash exercise Oulu^a^	
+ Plane crash exercise Kuusamo^a^	
Mexico	
+ Gas explosion	
Norway	
+ Bus rollover in Skaidi	
+ Truck and tunnel fire	
+ Utøya shootings	
+ Train collision	
United Kingdom	
+ Sheppy Crossing Bridge Road Traffic Accident	

^a^Indicates a major incident exercise

### Revising the template

A feasibility study conducted shortly after the website was launched identified that the template was too detailed and there was little relevance to some of the questions [13]. A revised, more user-friendly template was launched in December 2015 and a download pdf of the template was made available to give a more realistic view of the workload.

Several informants in the feasibility study also mentioned language as a barrier to completing the template. Efforts are underway to translate the template to several languages.

It is recognised that there is huge variability between countries when it comes to the emergency response to major incidents and the template was designed with developed countries in mind. However, it could be as applicable to under-developed counties and once we receive more reports we will be able to determine whether a separate or adjusted template needs to be implemented.

### Recruitment of reports

The ultimate aim of the database is to learn lessons that can be used to improve major incident response systems. The more reports that are published on the database, the more representative the analysis of reports will be.

In the two years since launching the database, 8 major incident reports and 2 major incident exercise reports have been published (Table 1). The steering group has identified a number of strategies to increase recruitment of reports to the database. As well as a more user-friendly template and website, increased publicity and understanding of the database is needed for it to succeed. In November, an animated video that explains the project, its conception, its importance and the process involved in submitting a report was published on the website (https://www.youtube.com/watch?v=tisymgIwpXY). The video has also been distributed to relevant parties, posted on social media and will be used to pitch for future funding and endorsement. Collaboration with like-minded, established organisations is an important step in the recruitment of reports.

As we are now accepting reports from major incident exercises, we have the potential to significantly increase the number of reports in a short period of time. In addition, using the template for reporting major incident exercises allows those using the template to familiarise themselves with it, thereby facilitating its use when real-life incidents occur.

Payment for performance is used successfully by many healthcare agencies to provide incentive for achieving certain goals. One example is the Trauma Audit Research Network (TARN) (https://www.tarn.ac.uk), which remunerates hospitals in England for submitting data on all severely injured trauma patients within 25 days of discharge or death. As a result, submission rates to TARN are high. A similar scheme could be introduced for MIR, especially if governments and local authorities become involved in the drive to collect major incident data.

### Data protection

The author of each report is solely responsible for ensuring that it does not contain any sensitive or confidential information and that it complies with ethical and privacy legislation. These issues can be daunting for authors, especially if something went wrong during the response. A drive towards a more open culture that reduces individual blame and looks for system errors should be encouraged.

The Norwegian Data Protection Authority granted approval for the database prior to its launch. One caveat was that no data on less than 6 patients be submitted to the database as this may allow identification of individuals. There are a number of disadvantages to this rule and further study will be made into data protection regulations to make it easier for authors and to improve accuracy of the data.

### Research projects

Finnish colleagues have used the Barents Rescue field exercise to conduct an inter-rater variability study to investigate whether or not several teams reporting from the same incident will gather identical data [14]. This will be an important first step in validating the content of the template.

South American colleagues are currently carrying out a feasibility study interviewing people responsible for major incident management to identify important data variables deemed essential when reporting on major incidents.

A recent systematic review indicated a need for more knowledge on the role of Helicopter Emergency Medical Services (HEMS) in major incidents [15]. This lead to a consensus process being undertaken to create a HEMS major incident reporting template [16]. This template is also now available on majorincidentreporting.net.

## Conclusion

Creating a database of major incident reports is just the beginning. In order to build a meaningful and implementable source of knowledge and an instrument for change to major incident response, the database must be recognised across emergency response agencies as a comprehensive, authoritative, and reliable source of information and ideas. To do this, it must gain backing from already established and trusted organisation and be widely publicised and supported by evidence-based research to prove its benefit. We welcome colleagues in the emergency and disaster medicine community to support us in these efforts.
